# H_2_S regulates endothelial nitric oxide synthase protein stability by promoting microRNA-455-3p expression

**DOI:** 10.1038/srep44807

**Published:** 2017-03-21

**Authors:** Xing-Hui Li, Wen-Long Xue, Ming-Jie Wang, Yu Zhou, Cai-Cai Zhang, Chen Sun, Lei Zhu, Kun Liang, Ying Chen, Bei-Bei Tao, Bo Tan, Bo Yu, Yi-Chun Zhu

**Affiliations:** 1 Research Center on Aging and Medicine, Fudan University, Shanghai Key Laboratory of Bioactive Small Molecules, Department of Physiology and Pathophysiology, Shanghai Medical College, Fudan University, Shanghai, China; 2 Department of physiology, Hainan Medical College, Haikou, Hainan 571101, China; 3 Department of Vascular Surgery, Huashan Hospital, Fudan University, Shanghai, 200040, China; 4 Department of Clinical Pharmacology, Shuguang Hospital, Affiliated to Shanghai University of Traditional Chinese Medicine, Shanghai, China

## Abstract

The aims of the present study are to determine whether hydrogen sulfide (H_2_S) is involved in the expression of endothelial nitric oxide synthase (eNOS) and nitric oxide (NO) production, and to identify the role of microRNA-455-3p (miR-455-3p) during those processes. In cultured human umbilical vein endothelial cells (HUVECs), the expression of miR-455-3p, eNOS protein and the NO production was detected after administration with 50 μM NaHS. The results indicated that H_2_S could augment the expression of miR-455-3p and eNOS protein, leading to the increase of NO level. We also found that overexpression of miR-455-3p in HUVECs increased the protein levels of eNOS whereas inhibition of miR-455-3p decreased it. Moreover, H_2_S and miR-455-3p could no longer increase the protein level of eNOS in the presence of proteasome inhibitor, MG-132. *In vivo*, miR-455-3p and eNOS expression were considerably increased in C57BL/6 mouse aorta, muscle and heart after administration with 50 μmol/kg/day NaHS for 7 days. We also identified that H_2_S levels and miR-455-3p expression increased in human atherosclerosis plaque while H_2_S levels decreased in plasma of atherosclerosis patients. Our data suggest that the stability of eNOS protein and the NO production could be regulated by H_2_S through miR-455-3p.

Hydrogen sulfide (H_2_S), a well-known toxic gas, is generated endogenously from L-cysteine by pyridoxal-5′-phosphate-dependent enzymes, including cystathionine γ-lyase (CSE), cystathionine β-synthase (CBS) and the zinc-dependent enzyme 3-mercaptopyruvate sulphur transferase (3-MST)^1^,^2^,^3^,^4^. H_2_S is a weak acid in aqueous solution, it can dissociate into H^+^ and hydrosulfide anion (HS^−^), which in turn may dissociate to H^+^ and sulfide anion (S^2−^)^5^. NO was first defined as an endothelial-derived relaxing factor, and is found to be highly relevant to cardiovascular health^6^. eNOS and inducible NOS (iNOS) are both expressed in the vascular endothelial cells, they are main origins of the vascular NO and play important roles in the regulation of angiogenesis and the pathogenesis of atherosclerosis^7^,^8^,^9^,^10^. The biological profiles of H_2_S and NO are similar, but the integrated vascular effects of these two gasotransmitters are quite complex. Previous researches attended to consider the short-term effects of H_2_S on NO production. Altaany *et al*. have shown that H_2_S therapy augment NO production, their results showed that H_2_S can increase the coupling of eNOS by inducing S-sulfhydration and inhibiting S-nitrosylation, which leads to an increased activity of eNOS. H_2_S also increase eNOS activity through promoting its phosphorylation^11^. On the contrary, high concentration of NaHS (300–3000 μM) significantly inhibited the activity of recombinant bovine eNOS^12^. Hu *et al*. reported synthetic H_2_S-NO hybrid molecule exhibited significantly greater potency of pro-angiogenic than that of H_2_S and/or NO donor alone^13^. Furthermore, the mechanisms by which H_2_S regulates eNOS remain to be clarified.

MicroRNAs (miRNAs) are a class of small, noncoding RNAs containing about 22 nucleotides that regulate gene expression through induction of mRNA degradation or translational repression. Our previous microarray data has shown that H_2_S could increase the level of miR-455-3p in HUVECs^14^. Plasma miR-455-3p has been reported to serve as a potential biomarker for abdominal aortic aneurysm^15^. Min *et al*. found that the expression of miR-455-3p was associated with cartilage development in bone matrix gelatin (BMG) rat model^16^. Several predicted target genes of miR-455-3p such as DNAJB12 (DnaJ heat shock protein family (Hsp40) member B12), DNAJB14 (DnaJ heat shock protein family (Hsp40) member B14), HERPUD1 (homocysteine-inducible, endoplasmic reticulum stress-inducible, ubiquitin-like domain member 1) as well as CUL3 (Cullin3) are related to proteasomal degradation. DNAJB12 and DNAJB14 belong to the HSP40 protein family, which has been shown to facilitate the proteasomal degradation of neuronal nitric oxide synthase (nNOS) through enhancing their ubiquitination^17^. CUL3 has been reported to be associated with other proteins to form ubiquitin ligase complex and participated in the Ubiquitylation and degradation of proteins^18^. Since eNOS expression could also be regulated through proteasomal degradation pathway, we hypothesize that H_2_S regulates proteasomal degradation of eNOS protein by manipulating miR-455-3p expression.

Angiogenesis is a process during which new capillaries form from existing ones and migration of endothelial cells plays a vital role during this progress. Atherosclerosis is a common disease that stems from the accumulation of fatty/cholesterol plaques under the endothelial barriers. H_2_S has been recognized as a gasotransmitter that regulates angiogenesis and atherosclerosis both *in vitro* and *in vivo*^19^,^20^,^21^. To investigate whether miR-455-3p is a regulator of eNOS during the regulation of endothelial cell migration and atherosclerosis plaque formation could elucidate the mechanisms by which H_2_S exerts its beneficial effects on endothelium protection and provide experimental data for further clinical applications of H_2_S derivatives.

In the present study, we examined if miR-455-3p was involved in the process of H_2_S regulated eNOS stability. We also collected atherosclerosis plaques from patients to test the relevance among endogenous H_2_S, miR-455-3p and eNOS.

## Results

### H_2_S promotes miR-455-3p and eNOS expression in HUVECs

**Figure 1:**
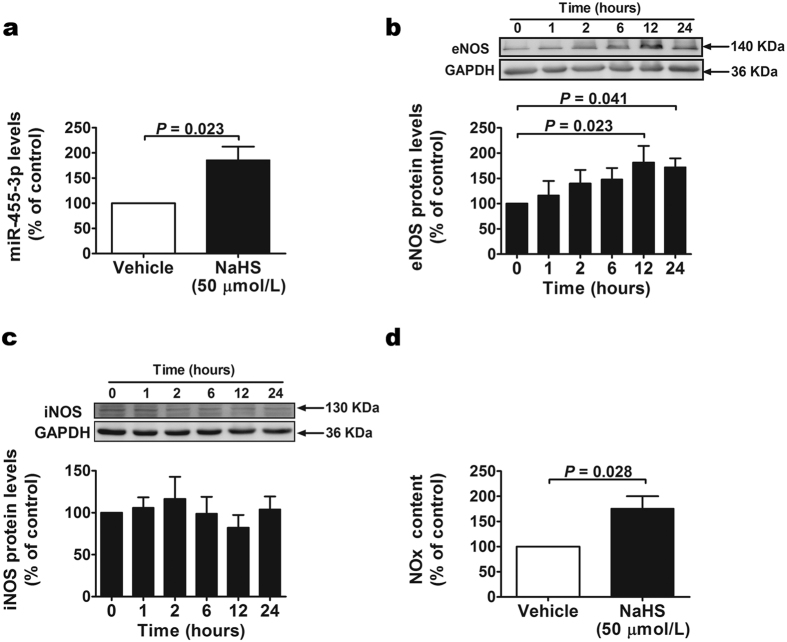
The effects of exogenous H_2_S on miR-455-3p, eNOS and iNOS expression in HUVECs. (**a**) The expression of miR-455-3p was increased in HUVECs treated with 50 μM NaHS for 24 h. Data represent the mean ± SE of six individual experiments. (**b**) The protein levels of eNOS were increased in HUVECs treated with 50 μM NaHS. Data represent the mean ± SE of six individual experiments. (**c**) The protein levels of iNOS did no change in HUVECs treated with 50 μM NaHS. Data represent the mean ± SE of six individual experiments. (**d**) NO production was increased in HUVECs treated with 50 μM NaHS for 24 h. Data represent the mean ± SE of six individual experiments.

Cultured HUVECs were harvested after treated with vehicle or NaHS for 24 h. Then total RNA was extracted and real-time PCR experiments were performed to determine the expression of miR-455-3p. As shown in Fig. 1a compared with vehicle group, the expression of miR-455-3p was increased with NaHS administration. To determine the effects of H_2_S on eNOS and iNOS expression, proteins were extracted from HUVECs at 0, 1, 2, 6, 12 and 24 h after NaHS administration. Western blots showed that H_2_S increased eNOS expression at 12 and 24 h (Fig. 1b), but has no effect on iNOS expression (Fig. 1c). Our data also showed that H_2_S promoted NO production in HUVECs after 24 h exposure to NaHS (Fig. 1d).

### miR-455-3p mediates H_2_S induced eNOS expression

**Figure 2:**
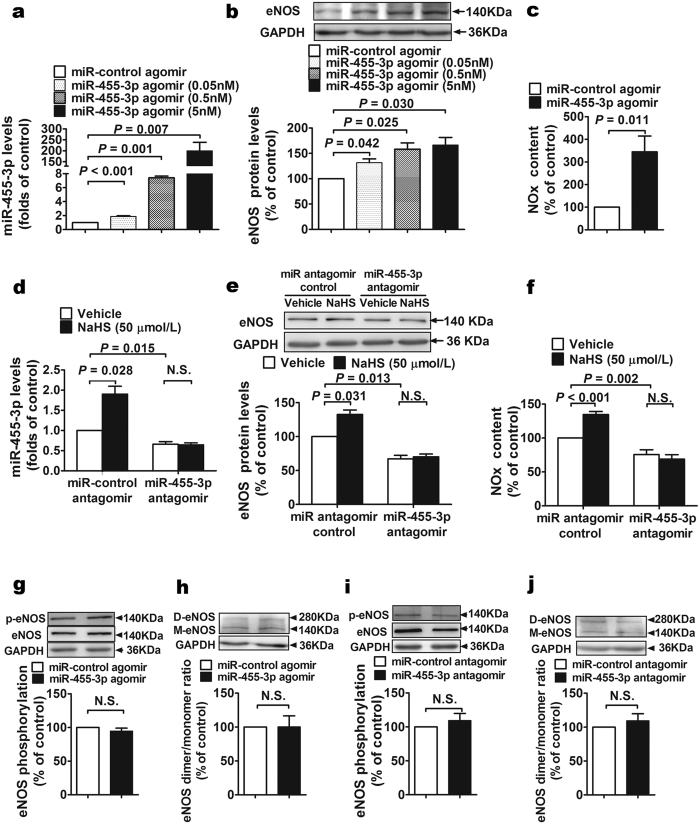
The effects of miR-455-3p on eNOS expression, NO production and eNOS coupling. (**a**) The expression of miR-455-3p was increased in HUVECs transfected with miR-455-3p agomir (0.05 nM, 0.5 nM and 5 nM) compared with control. Data represent the mean ± SE of five individual experiments. (**b**) The protein levels of eNOS increased after miR-455-3p agomir (0.05 nM, 0.5 nM and 5 nM) transfection compared with control. Data represent the mean ± SE of six individual experiments. (**c**) NO production was increased in HUVECs transfected with 5 nM miR-455-3p agomir compared with control. Data represent the mean ± SE of six individual experiments. (**d**) The expression of miR-455-3p was decreased in HUVECs transfected with 150 nM miR-455-3p antagomir compared with control. H_2_S induced increasing expression of miR-455-3p were blocked when the expression of miR-455-3p were inhibited. Data represent the mean ± SE of six individual experiments. (**e**) Transfection of 150 nM miR-455-3p antagomir decreased the protein levels of eNOS in HUVECs and blocked the promotion effect of H_2_S on eNOS protein levels. Data represent the mean ± SE of five individual experiments. (**f**) Transfection of 150 nM miR-455-3p antagomir decreased the NO production in HUVECs and blocked the promotion effect of H_2_S on NO production. Data represent the mean ± SE of seven individual experiments. (**g,h**) Transfection of 5 nM miR-455-3p agomir did not change the phosphorylation and coupling (indicated by the ratio of dimer to monomer) levels of eNOS compared with control. Data represent the mean ± SE of six individual experiments. (**I,j**) Transfection of 150 nM miR-455-3p antagomir had no effect on the phosphorylation and the coupling of eNOS. Data represent the mean ± SE of six individual experiments.

As shown in Fig. 2a, HUVECs transfected with 5 nM miR-455-3p agomir showed an increased expression of miR-455-3p for nearly 200-folds. When we decreased the dosage of miR-455-3p agomir to 0.5 nM or 0.05 nM, the expression level of miR-455-3p increased 8 or 2 folds respectively. HUVECs with all three dosages of miR-455-3p overexpression have elevated eNOS expression (Fig. 2b). 5 nM miR-455-3p agomir was chosen to perform the following experiments since it caused highest eNOS expression. Firstly, we examined NO production after miR-455-3p overexpression. Elevated miR-455-3p caused increased NO production (Fig. 2c). Secondly, inhibiting expression of miR-455-3p to 50% (Fig. 2d) leaded to decreased expression of eNOS protein and NO content (Fig. 2e,f). Interestingly, the effects of H_2_S on eNOS expression and NO production were blocked by miR-455-3p inhibition (Fig. 2e,f). As shown in Fig. 2g,h, overexpression of miR-455-3p (5 nM) has no effects on phosphorylation or coupling levels of eNOS. Figure 2i,j showed that inhibiting expression of miR-455-3p has no effects on phosphorylation or coupling levels of eNOS. Our results also showed that there is no change in iNOS expression either in miR-455-3p overexpressed cells or in downregulated ones (Supplementary Figure S1a and 1b).

### H_2_S and miR-455-3p downregulate the proteasome degradation pathway of eNOS protein

**Figure 3:**
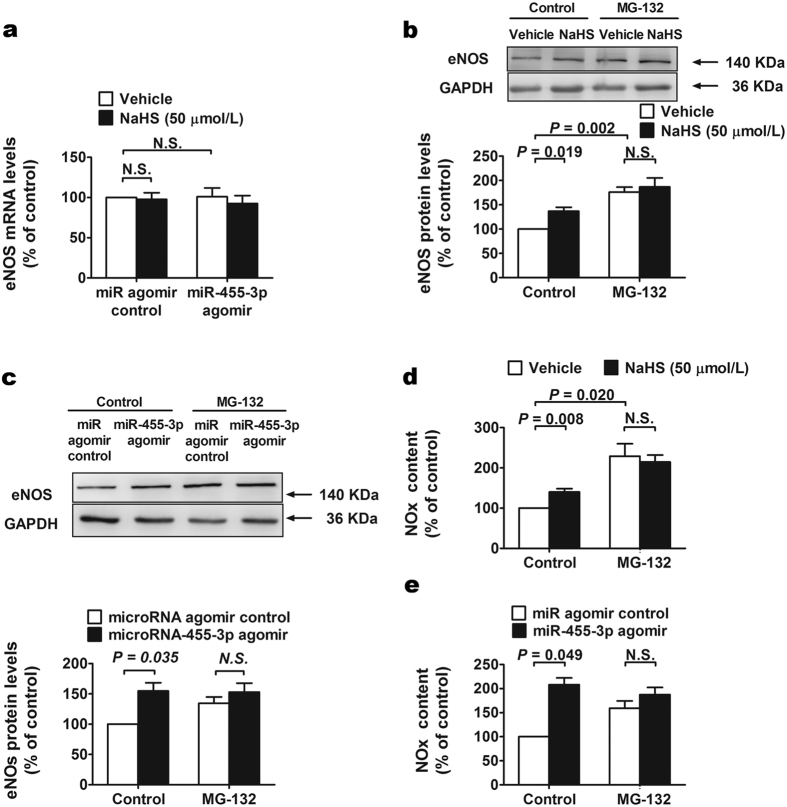
Ubiquitin-proteasome pathway inhibitor MG-132 blocked the effects of H_2_S and miR-455-3p on eNOS expression. (**a**) 50 μM NaHS administration for 24 h or overexpression of miR-455-3p with 5 nM agomir did not change the mRNA levels of eNOS. Data represent the mean ± SE of seven individual experiments. (**b**) MG-132 (1uM) added for 12 h before cell lysis blocked the promotion effect of H_2_S on eNOS expression. Data represent the mean ± SE of seven individual experiments. (**c**) MG-132 (1 uM) added for 12 h before cell lysis blocked the promotion effect of miR-455-3p on eNOS expression. Data represent the mean ± SE of seven individual experiments. (**d**) MG-132 (1 uM) added for 12 h before cell lysis blocked the promotion effect of H_2_S on NO production. Data represent the mean ± SE of eight individual experiments. (**e**) MG-132 (1 uM) added for 12 h before cell lysis blocked the promotion effect of miR-455-3p on NO production. Data represent the mean ± SE of six individual experiments.

The mRNA levels of eNOS were detected by Real-time PCR and the results showed that there was no significant change either by NaHS treatment or miR-455-3p agomir transfection (Fig. 3a). However, eNOS protein level (Fig. 3b,c) and NO content (Fig. 3d,e) increased after H_2_S administration and miR-455-3p transfection. Our results indicate the post-transcriptional regulation of NO production by H_2_S and miR-455-3p. We found that proteasome degradation pathway of eNOS is regulated by H_2_S and miR-455-3p. Both H_2_S and miR-455-3p induced eNOS expression (Fig. 3b,c) and NO production (Fig. 3d,e) were blocked with the presence of MG-132 (1 μM).

### CUL3 is the target gene of miR-455-3p in HUVECs

**Figure 4:**
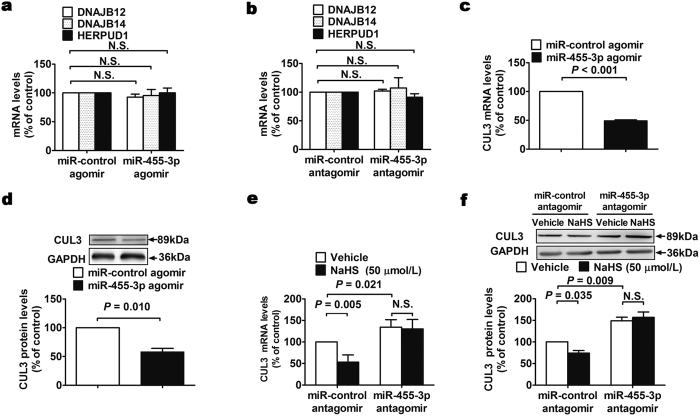
CUL3 is the target gene of miR-455-3p. (**a,b**) Transfection of 5 nM miR-455-3p agomir or 150 nM antagomir did not change the mRNA levels of DNAJB12, DNAJB14 and HERPUD1 compared with control. Data represent the mean ± SE of six individual experiments. (**c**) The mRNA levels of CUL3 decreased after 5 nM miR-455-3p agomir transfected compared with control. Data represent the mean ± SE of six individual experiments. (**d**) The protein levels of CUL3 decreased after 5 nM miR-455-3p agomir transfected compared with control. Data represent the mean ± SE of six individual experiments. (**e**) Transfection of 150 nM miR-455-3p antagomir increased the mRNA levels of CUL3 and blocked the inhibition effect of H_2_S on CUL3 mRNA levels. Data represent the mean ± SE of six individual experiments. (**f**) Transfection of 150 nM miR-455-3p antagomir increased the protein levels of CUL3 and blocked the inhibition effect of H_2_S on CUL3 protein levels. Data represent the mean ± SE of six individual experiments.

miRecords predicted several target genes of miR-455-3p such as DNAJB12, DNAJB14, HERPUD1 as well as CUL3 which are all related to proteasome degradation (Supplementary Table S1). DNAJB12, DNAJB14, HERPUD1 mRNA expression did not change either in miR-455-3p overexpressed or in miR-455-3p downregulated HUVECs (Fig. 4a,b). Interestingly, we found that HUVECs with overexpressed miR-455-3p have decreased CUL3 expression both in mRNA and protein levels (Fig. 4c,d). Furthermore, 50 μM NaHS decreased the expression of CUL3 in mRNA and protein levels (Fig. 4e,f). Inhibiting expression of miR-455-3p not only leaded to increased expression of CUL3 but also blocked the effects of NaHS on CUL3 production (Fig. 4e,f).

### H_2_S promotes migration of HUVECs through increased expression of eNOS and miR-455-3p

**Figure 5:**
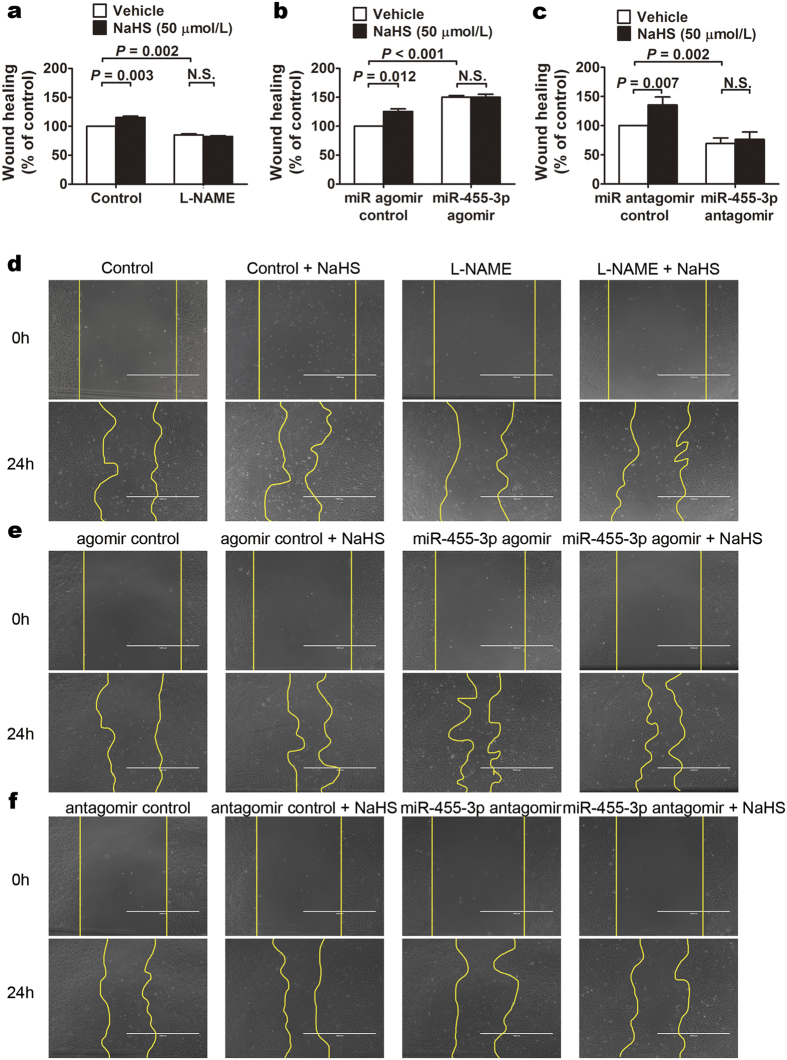
The role of eNOS and miR-455-3p during H_2_S-mediated migration of HUVECs. (**a**) 50 μM NaHS administration for 24 h increased the migration of HUVECs and the effects of H_2_S-mediated migration were blocked when treated with eNOS inhibitor, L-NAME(100 μM). Data represent the mean ± SE of six individual experiments. (**b**) Overexpression of miR-455-3p with 5 nM miR-455-3p agomir increased the migration of HUVECs and the effects of H_2_S-mediated migration were blocked. Data represent the mean ± SE of six individual experiments. (**c**) Downregulation of miR-455-3p with 150 nM miR-455-3p antagomir decreased the migration of HUVECs and blocked the effects of H_2_S-mediated migration. Data represent the mean ± SE of six individual experiments. d, e and f are representative photomicrographs of a, b and c respectively. Scale bar = 1000 μm.

Wound healing assay was chosen to evaluate endothelial cell migration. eNOS inhibitor L-NAME (100 μM) was used to inhibit NO production in HUVEC for 35% without changing the expression of iNOS expression (Supplementary Figure S1c and 1d). The migration of HUVECs was obviously blocked in L-NAME pre-treated group, and the promoting effect of H_2_S on migration was also abolished, indicating the necessity of endogenous NO in this process (Fig. 5a,d). To investigate the role of miR-455-3p in HUVEC migration, miR-455-3p overexpression was achieved by agomir transfection. As demonstrated in Fig. 5b,e, HUVECs overexpressing miR-455-3p showed a remarkable increase of migration when compared to the control groups. Conversely, when endogenous miR-455-3p expression was inhibited by miR-455-3p antagomir, cell migration was blunted (Fig. 5c,f). Interestingly, in both overexpression and inhibition cases, the pro-migration effect of H_2_S was concealed (Fig. 5b,c,e,f).

### eNOS and miR-455-3p expression increased in mice tissues after H_2_S administration

**Figure 6:**
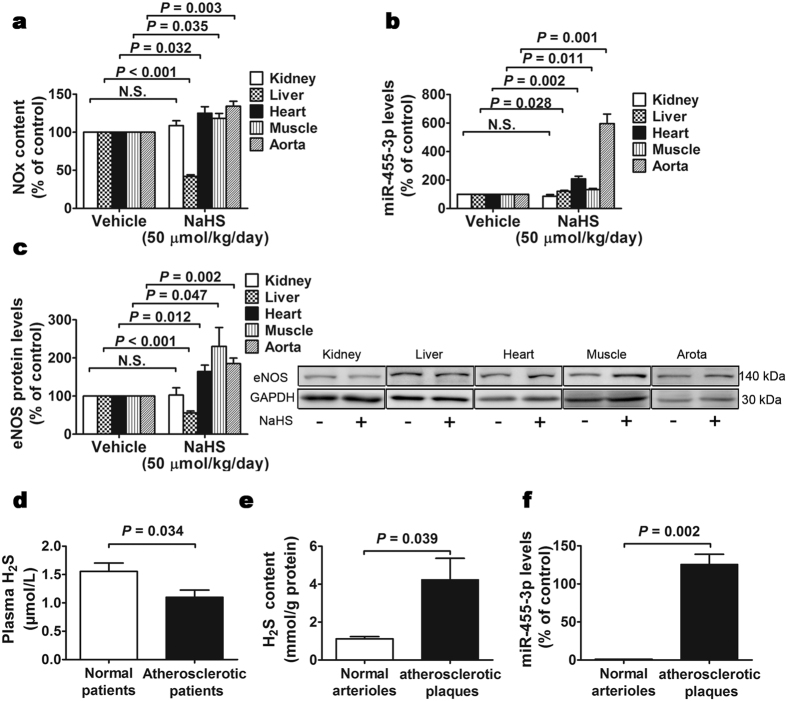
The effects of exogenous H_2_S on the expression of eNOS, miR-455-3p and NO production in mice, and comparison of miR-455-3p expression and H_2_S production between atherosclerosis patients and chronic venous insufficiency patients. (**a**) Intraperitoneal injection of 50 μmol/kg/day NaHS for 7 days increased NO production in heart, skeletal muscles and aorta compared with vehicle. Meanwhile, NO production in liver was decreased. No significant change was observed in kidney NO production. Data represent the mean ± SE of six animals in each group. (**b**) 50 μmol/kg/day NaHS administration increased miR-455-3p expression in heart, skeletal muscles, aorta and liver. No significant change was observed in kidney miR-455-3p expression. Data represent the mean ± SE of six animals in each group. (**c**) Protein levels of eNOS did not change significantly in kidney, but increased in heart, skeletal muscle and aorta after 50 μmol/kg/day NaHS administration as compared with Vehicle. Conversely, eNOS protein levels decreased in liver. Data represent the mean ± SE of six animals in each group. (**d**) H_2_S levels in plasma reduced in atherosclerotic patients compared with control patients. Data represent the mean ± SE of eight normal patients and nine atherosclerotic patients. (**e**) H_2_S levels in atherosclerotic plaques increased compared with normal arterioles. Data represent the mean ± SE of five patients in each group. (**f**) Expression of miR-455-3p increased in atherosclerotic plaques compared with normal arterioles. Data represent the mean ± SE of five patients in each group.

To investigate if H_2_S could influence miR-455-3p and eNOS expression *in vivo*, 8-week-old mice were injected intraperitoneally with normal saline or 50 μmol/kg/day NaHS for one week. Our results showed that eNOS protein levels, miR-455-3p levels as well as NO content did not change in kidney tissues (Fig. 6a–c). Our results also showed that NO production and eNOS protein levels decreased in liver tissues (Fig. 6a,c) while miR-455-3p increased in liver tissues (Fig. 6b) after NaHS administration. Nevertheless, exogenous H_2_S remarkably increased both NO content (Fig. 6a) and miR-455-3p expression (Fig. 6b) in heart, muscle and aorta tissues. Changes in miR-455-3p expression is more profound than NO content. While the increase range of NO content is only 10 to 30 percent, miR-455-3p expression increased in heart tissues for 2-fold, muscle tissues for 1.5-fold and aorta tissues for nearly 6-folds (Fig. 6a,b). In heart, muscle and aorta tissues, the elevated NO content is at least partially due to upregulated eNOS levels (Fig. 6a–c).

### H_2_S and miR-455-3p increased in atherosclerotic plaques compared with normal arterioles

Plasma H_2_S levels in carotid arterial stenosis patients (atherosclerosis group) and chronic venous insufficiency patients (control group) were measured and our data showed that H_2_S content in plasma were significantly decreased in atherosclerosis patients compared with control patients (Fig. 6d). However, local H_2_S levels in atherosclerotic plaques increased when compared with normal arterioles (Fig. 6e). We also detected the expression of miR-455-3p in those patients and found that miR-455-3p levels increased in atherosclerotic plaques when compared with normal arterioles (Fig. 6f).

## Discussion

H_2_S has been found to be involved in many physiological and pathophysiological processes through anti-apoptotic, anti-inflammatory, anti-hypertrophic, cardioprotective, and anti-oxidant effects^22^. Our previous studies reported a pro-angiogenic role of H_2_S on HUVECs in physiological/pathophysiological models through targeting VEGFR2^19^,^23^. Recently, we also reported that the expression of 12 miRNAs in HUVECs were changed after administration of NaHS using an Affymetrix gene chip expression assay. Among these miRNAs, miR-640 is down-regulated and regulates the stability of hypoxia-inducible factor 1 alpha (HIF1A) mRNA^14^. Considerable evidence has been provided to support the central role of eNOS in regulating endothelial cell functions such as migration^24^. It has been reported that a high concentration of NaHS (300–3000 μM) significantly inhibited the activity of recombinant bovine eNOS^13^. Conversely, Altaany *et al*. demonstrated that NaHS (50 and 100 μΜ) treatment for 30 min promoted NO production in human umbilical vein endothelial cells-derived EA.hy 926 cells by stimulating the phosphorylation of eNOS without changing its expression^25^. In the present study, we confirmed that NaHS (50 μΜ) treatment promotes NO production in HUVECs. This effect is mediated by increased eNOS expression, but not iNOS protein levels. We also found that increased expression of miR-455-3p was involved in NaHS induced eNOS expression, NO production, and HUVEC migration.

In the current study, we confirmed the results that eNOS plays a vital role in pro-migration effect of H_2_S^26^. We identified for the first time that upregulation of miR-455-3p expression could promote the migration of HUVECs while downregulation of miR-455-3p could inhibit the migration. Inhibition of miR-455-3p also blocked the pro-migration effect of H_2_S. These results indicate that miR-455-3p played an important role in H_2_S and eNOS mediated migration of HUVECs, and involves in the regulation of NO production.

To investigate the mechanism of NO production regulation, we transfected agomir or antagomir of miR-455-3p in HUVECs to study its effect on eNOS expression, phosphorylation as well as the coupling of eNOS. As shown by our results, overexpression of miR-455-3p dramatically elevated the protein levels of eNOS and NO content in HUVECs. Moreover, downregulation of miR-455-3p attenuated eNOS expression and NO content in HUVECs. We also found that the promoting effect of H_2_S on eNOS expression was abolished by the inhibition of miR-455-3p. However, neither overexpression nor downregulation of miR-455-3p has any effect on phosphorylation or dimerization of eNOS. So that, we speculated that the stability of eNOS might be regulated by miR-455-3p. Proteasome inhibitor MG-132 was used to detect whether miR-455-3p is involved in the ubiquitination and degradation of eNOS or not. After adding MG-132 to HUVECs, we found that the promoting effect of H_2_S and miR-455-3p on eNOS expression was abolished. This result indicates that H_2_S enhances NO production by inhibiting proteasome degradation of eNOS. To explore the mechanism of miR-455-3p on eNOS expression, we first identified potential targets for miR-455-3p by employing database miRecords (http://c1.accurascience.com/miRecords/). miRecords is an integration of predicted miRNA targets produced by 11 established miRNA target prediction programs. Several predicted target genes of miR-455-3p such as DNAJB12, DNAJB14, HERPUD1 as well as CUL3 are related to proteasome degradation (Supplementary Table S1). CUL3 could associate with other proteins to form ubiquitin ligase complex^18^. Real-time PCR and Western blot results showed that CUL3 is the target gene of miR-455-3p.Hydrogen sulfide increases miR-455-3p, thus downregulates CUL3 and inhibits the proteasome degradation of eNOS protein.

To further investigate *in vivo* effects of H_2_S on miR-455-3p and their potential effects on the pathogenesis of diseases, we detected the expression of miR-455-3p in mouse healthy tissues. We found that H_2_S administration increased NO production in heart, muscle and aorta, while decreased in liver. NO production did not change in kidney with H_2_S treatment. Western blots and Real-time PCR results showed that NaHS administration increased the expression of miR-455-3p and eNOS protein levels in skeletal muscle, heart and aorta. miR-455-3p and eNOS protein levels in kidney did not change after NaHS administration. In liver, miR-455-3p levels increased while eNOS protein levels and NO production decreased. Gopi K *et al*. reported that exogenous H_2_S increased NO production in mouse by activating eNOS in the skeletal muscle during hind limb ischaemia^27^. Benjamin L *et al*. reported preservation of endogenous H_2_S protects the ischemic myocardium by increasing NO bioavailability through eNOS phosphorylation at Ser1177^28^. We speculate that there are variety pathways to regulate the expression of eNOS *in vivo*, specific organs may employ different mechanisms to regulate local NO production. In heart, muscle and aorta, miR-455-3p seems to play a vital role in eNOS regulation, while in kidney and liver, it does not play a decisive role. We also speculate that in some tissues H_2_S regulates NO production not only by promoting eNOS protein expression but also by increasing its stability.

A number of studies looked into the use of NO and H_2_S as a marker of cardiovascular diseases in humans, such as the early development and progression of atherosclerosis^7^,^29^. eNOS-derived NO possess multiple anti-atherosclerotic properties. Under conditions of atherosclerosis and vascular disease, NO bioavailability in the vasculature is reduced because of eNOS uncoupling and reduced eNOS activity, however, eNOS expression could be compensatorily enhanced during those processes^30^,^31^. Muzaffar *et al*. reported that H_2_S could attenuate the progress of atherogenesis by inhibiting superoxide formation in the early phase of plaque development^32^. Although a protective role of H_2_S against atherosclerosis has been recognized, mechanism underlying the anti-atherosclerotic effect of H_2_S need to be settled and the therapeutic value of H_2_S towards atherosclerosis need to be tested clinically. J. C. van *et al*. demonstrated that intraplaque H_2_S production could aggravate plaque vulnerability by promoting intraplaque angiogenesis^33^. Therefore, we collected some normal arterioles and atherosclerotic plaques from patients to investigate if H_2_S and miR-455-3p level changes and participate in the reduced NO synthesis in the plaque. Firstly, we confirmed that H_2_S level decreased in plasma from atherosclerosis patients compared with patients without atherosclerosis^34^ (here we use plasma from chronic venous insufficiency patients as control). However, the tissue level of H_2_S and miR-455-3p increased in atherosclerotic plaques compared with normal arterioles. Our results indicate that H_2_S and miR-455-3p may participate in the compensation mechanism of eNOS expression in atherosclerotic plaque. However, the number of human samples is small in our experiments, more clinical samples and animal studies are needed to further investigate whether the compensation effect on NO production during atherosclerotic plaque formation is caused by increased H_2_S concentration and miR-455-3p expression.

Taken together, the current work discovered for the first time that miR-455-3p was involved in the pro-migration effect of H_2_S on endothelial cells and mediates the effect of H_2_S on eNOS protein stability through ubiquitination pathway. H_2_S may also participate in the compensation mechanism of eNOS expression in atherosclerotic plaque.

## Methods

### Cells Culture

Primary human umbilical vein endothelial cells were purchased from Allcells (Shanghai, China) and cultured in endothelial complete medium (ECM; Allcells, China) as described previously^21^. HUVECs were digested and plated onto 3.5 cm culture dishes coated with 0.5% gelatin for the following experiments. Only cells from four to seven passages were used, and each experiment has been repeated from different batches of HUVECs. HUVECs were starved for 12 hours with basal medium (EBM with 1% FBS; Allcells, China) before experiment. Basal medium was also used to culture HUVECs underwent experiments. NaHS was added to medium as H_2_S donor at final concentration of 50 μM and double distilled water served as vehicle control. N^G^-nitro-L-arginine methyl ester (L-NAME) (Beyotime, China, 100 μM) pre-treatment for 24 h was used to inhibit the activity of eNOS. MG-132 (Beyotime, China, 1uM) was used to block the ubiquitin-proteasome pathway. It was added to medium 12 h before cell lysis and Dimethylsulfoxide (DMSO) served as vehicle control.

### Synthesis and transfection of has-miR-455-3p agomir and antagomir

The has-miR-455-3p agomir oligonucleotide (5′-GCAGUCCAUGGGCAUAUACAC-3′), the has-miR-455-3p antagomir oligonucleotide (target sequence 5′-GUGUAUAUGCCCAUGGACUGC-3′), the agomir negative control and the antagomir negative control were synthesized by Biotend (Shanghai, China). HUVECs were transfected with 0.05 nM, 0.5 nM, 5 nM has-miR-455-3p agomir to overexpress miR-455-3p and 150 nM has-miR-455-3p antagomir to downregulate it. These oligonucleotides were transfected into HUVECs with Lipofectamine RNAiMAX Reagent (Invitrogen, USA) according to the manufacturer's protocol.

### Real-time PCR

Total RNA of HUVECs and animal tissues were extracted using TRIzol Reagent (Invitrogen, USA) according to the manufacturer’s protocol. Afterwards, 500 ng RNA from each sample was reverse transcribed into complementary DNA (cDNA) using a Reverse Transcription Kit (Tiangen, China). mRNA was reversed using Oligo-dT primer. miR-455-3p was reversed using stem-loop RT-PCR, reverse primer for miR-455-3p was 5′-GTCGTATCCAGTGCAGGGTCCGAGGTATTCGCACTGGATACGACGTGTAT-3′. RNU6B served as endogenous reference when detecting miR-455-3p and the primer sequence for RNU6B reversion was 5′-AACGCTTCACGAATTTGCGT-3′. Amplification and detection of cDNA were performed with the StepOnePlus™ Real-Time PCR System (Applied Biosystems, USA) using SYBRGreen RT-PCR Kit (Toyobo, Japan). The sequences for primers of Real-time PCR are summarized as follows: GAPDH (forward 5′-TGCCCCCATGTTCGTCA-3′ and reverse 5′-CTTGGCCAGGGGTGCTAA-3′), eNOS (forward 5′-ACCCTCACCGCTACAACAT-3′ and reverse 5′-GCCTTCTGCTCATTCTCCA-3′), CUL3 (forward 5′-TGTGGAGAACGTCTACAATTTGG-3′ and reverse 5′-GCGCCTCTGTCTACGACTT-3′), DNAJB12 (forward 5′-TTCCCTTCTAGTAACGTCCACG-3′ and reverse 5′-GCTGAGAGCTGACACGAGAAT-3′), DNAJB14 (forward 5′-ATGAGGCTGAGAAATGTGTCG-3′ and reverse 5′-GGTTTTCGGCAATGAGGGCTA-3′), HERPUD1 (forward 5′-ATGGAGTCCGAGACCGAAC-3′ and reverse 5′-TTGGTGATCCAACAACAGCTT-3′), has-miR-455-3p (forward 5′-TAAGACGTCCATGGGCAT-3′ and reverse 5′-GTGCAGGGTCCGAGGT-3′), mmu-miR-455-3p (forward 5′-TAAGACGTCCACGGGCAT-3′ and reverse 5′-GTGCAGGGTCCGAGGT-3′), RNU6B (forward 5′-CTCGCTTCGGCAGCACA-3′ and reverse 5′-AACGCTTCACGAATTTGCGT-3′).

### Scratch Wound Healing Assay

Confluent HUVECs were starved for 12 h before experiments to inhibit cell proliferation. 200 μL pipette tips were used to generate scratch wounds, then cells were rinsed once in Basal medium and photographed immediately. Those areas were photographed again after 24 h incubation with different treatments. EVOS digital inverted microscope (AMG, Seattle, USA) was used to take photograph and Image J software was used to analyze the wound area.

### SDS–PAGE and immunoblotting

HUVECs were lysed in RIPA lysis buffer (Beyotime, Jiangsu, China) and protein contents were determined using the BCA method. For normal SDS-PAGE, 30 ug protein samples were mixed with the sample loading buffer and boiled for 5 min. Then they were immediately loaded onto a 10–12% Tris-glycine polyacrylamide gradient gel. Protein Gels were run at room temperature for 0.5 h at 70 V for concentration and 1.5 h at 120 V for separation. For immunoblot analysis of the dimeric and monomeric form of eNOS protein, low-temperature SDS–PAGE(LT-PAGE) were used as described previously^35^,^36^. Gels and buffers were equilibrated at 4 °C before electrophoresis, and protein samples were mixed with native loading buffer. Then samples were loaded on 7.5% polyacrylamide gels and subjected to electrophoresis in an ice bath. Both SDS-PAGE and LT-PAGE were transferred to a PVDF membrane with 280 mA at 4 °C for 120 min. Antibodies against eNOS (mouse monoclonal antibodies, 1:2000; BD), iNOS (mouse monoclonal antibodies, 1:2000; CST), CUL3 (rabbit polyclonal antibodies, 1:1000; proteintech), GAPDH (rabbit polyclonal antibodies, 1:2000; proteintech) were used to detect specific proteins.

### Analysis of the intracellular nitric oxide production

HUVECs or tissues were lysed using RIPA lysis buffer and centrifuged at 12,000 g for 15 min at 4 °C to get cell suspension. The resultant lysates were then stored at −80 °C until NO detection. Total intracellular NO was measured using Total nitric oxide assay Kit (Beyotime, Jiangsu, China) by detecting 540 nm absorbance according to the manufacturer’s instructions. Protein contents were quantified using BCA method. NO levels were normalized to protein concentrations and compared with control groups.

### Measurement of H_2_S levels

H_2_S levels in tissues and plasma were measured according to the methods previously described^22^,^37^. Briefly, tissues were homogenized by ice-cold Tris-HCl (100 mmol/L, pH 8.5) and centrifuged at 12,000 g for 15 min at 4 °C. The resultant tissue lysates were immediately used to detect H_2_S levels. 10 μL Lysates were mixed with 80 μL monobromobimane (MBB, Sigma–Aldrich) for 1 hour at room temperature for derivatization of sulfide, which called sulfide-dibimane. The reaction was then terminated with 10 μL formic acid (30%) and centrifuged at 12,000 g for 15 min. The supernatants which contain sulfide-dibimane were stored at −80 °C until the detection of H_2_S levels by high-performance liquid chromatography (HPLC). H_2_S concentrations of tissues were normalized to the protein concentrations and compared with the control groups. H_2_S concentrations of plasma were compared to the control groups.

### Animals and treatments

12 female C57BL/6 mice, age 8 weeks were obtained from Slac (China) and raised in pathogen-free barrier facilities and monitored regularly by the veterinary staff. Animals were randomly divided into two groups, each group of animals were administered normal saline or 50 μmol/kg/day NaHS by intraperitoneal injection for 7 days. In this study, mice were sacrificed at day 7 by carbon dioxide narcosis and subsequent cervical dislocation. Then the heart, liver, kidney, hind limb muscles, and aorta of each mouse were dissected, snap frozen in liquid nitrogen, and stored at −80 °C until biochemical analyses. All animal experiments were performed in accordance with the Guide for the Care and Use of Laboratory Animals published by the NIH of the United States, and it was approved by the Ethics Committee for Experimental Research, Fudan University Shanghai Medical College. IACUC Animal Project Number (APN): 20120302-098.

### Sample Preparation from Patients

Atherosclerotic plaques were collected from carotid arterial stenosis patients who had hyperlipidemia caused atherosclerosis and underwent endarterectomy surgery. Small unnamed branches of superficial femoral artery were collected from chronic venous insufficiency patients underwent laser ablation surgery. After surgical removal, atherosclerotic plaques and arterial branch were snap-frozen in liquid nitrogen in the operating room. Venous blood was collected from those patients in lithium heparin vacutainer collection tubes 1 day before surgery. Informed consent was signed by each patient. Normal arterioles were used as control for atherosclerotic plaques. Patients underwent laser treatment of varicose veins of lower extremities were used as normal control for atherosclerotic patients. All methods were performed in accordance with the guidelines established by the Science Council of China. The study was approved by the Ethics Committee of Huashan Hospital, Fudan University. The approval number is 2016-224.

### Statistical analysis

The data were presented as mean ± SE of at least five separate experiments. Two treatment groups were compared using the Student′s t-test (SPSS Inc.). Multiple group comparisons were tested by one-way ANOVA (post-hoc analysis). Probability value less than 0.05 was considered statistically significant.

## Additional Information

**How to cite this article**: Li, X.-H. *et al*. H_2_S regulates endothelial nitric oxide synthase protein stability by promoting microRNA-455-3p expression. *Sci. Rep.* **7**, 44807; doi: 10.1038/srep44807 (2017).

**Publisher's note:** Springer Nature remains neutral with regard to jurisdictional claims in published maps and institutional affiliations.

## Supplementary Material

Supplementary Information

Supplementary Table S1
