# Supplementary material for: H2S regulates endothelial nitric oxide synthase protein stability by promoting microRNA-455-3p expression
Source: Sci Rep. 2017 Mar 21;7:44807. doi: 10.1038/srep44807 (PMC5359669; doi:10.1038/srep44807)
Supplement: Supplementary Information [file srep44807-s1.pdf]

H_2S regulates endothelial nitric oxide synthase protein stability by promoting microRNA-455-3p expression

Xing-Hui Li[1,†], Wen-Long Xue[1,†], Ming-Jie Wang[1,†], Yu Zhou[1], Cai-Cai Zhang[1,2], Chen Sun[1], Lei Zhu[3], Kun Liang[3], Ying Chen[1], Bei-Bei Tao[1], Bo Tan[4], Bo Yu[3], and Yi-Chun Zhu[1,*]

Supplementary Table S1 Legend

Supplementary Table S1 contains pool of data downloaded from miRecords, which is an integration of predicted miRNA targets produced by 11 established miRNA target prediction programs. We had listed some target genes of interest from this excel worksheet in our manuscript and these target genes are highlighted in yellow in the worksheet.

Supplementary Figure S1 and the Legend

(a) iNOS protein levels showed no significant change after transfected with 5nM miR-455-3p agomir compared with control. Data represent the mean ± SE of six individual experiments. (b) iNOS protein levels had no difference after 150nM miR-455-3p antagomir or 50 μM NaHS treatment for 24h compared with control. Data represent the mean ± SE of five individual experiments. (c) eNOS inhibitor L-NAME (100 μM, 24h) decreased NO production in HUVECs. Data represent the mean ± SE of five individual experiments. (d) Neither 50 μM NaHS nor 100 μM L-NAME administration for 24h had any effect on the levels of iNOS protein. Data represent the mean ± SE of six individual experiments

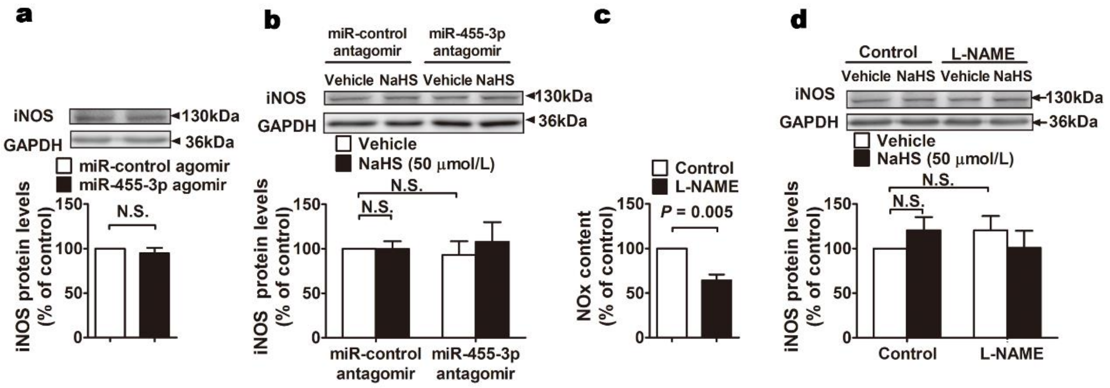
a
iNOS
130kDa
GAPDH
36kDa
miR-control agomir
miR-455-3p agomir
iNOS protein levels (% of control)
N.S.
b
miR-control antagomir
miR-455-3p antagomir
Vehicle
NaHS
iNOS
GAPDH
NaHS (50 μmol/L)
iNOS protein levels (% of control)
c
Control
L-NAME
P = 0.005
NOx content (% of control)
d
N.S.
